# The Proliferation Capacity of Cultured Neural Stem Cells Promoted by CSF Collected from SAH Patients Correlates to Clinical Outcome

**DOI:** 10.1038/s41598-018-19371-5

**Published:** 2018-01-18

**Authors:** Yun-An Chen, Kuo-Chuan Wang, Der-Zen Liu, Tai-Horng Young, Li-Kai Tsai

**Affiliations:** 1 Institute of Biomedical Engineering, College of Medicine and College of Engineering, National Taiwan University, Taipei, Taiwan; 2 Department of Surgery, National Taiwan University Hospital and National Taiwan University College of Medicine, Taipei, Taiwan; 3 Graduate Institute of Biomedical Materials and Tissue Engineering, College of Biomedical Engineering, Taipei Medical University, Taipei, Taiwan; 4 Department of Neurology and Stroke Center, National Taiwan University Hospital and National Taiwan University College of Medicine, Taipei, Taiwan

## Abstract

Neurogenesis from endogenous neural stem cells (NSCs) might contribute to functional recovery after stroke based on animal studies; however, the relationship between neurogenesis and post-stroke outcome has rarely been demonstrated in humans. We prospectively collected cerebrospinal fluid (CSF) from 36 patients with subarachnoid hemorrhage (SAH). The CSF was added to the culture medium of the rat NSCs to test the effects on proliferation (proliferation index [PI], percentage of Ki-67 immunoreactive cells). We correlated the PI with functional outcome based on the modified Rankin Scale at 3 months post-SAH. Treatment with the CSF samples collected from SAH patients showed a higher PI compared with those collected from patients with normal pressure hydrocephalus and untreated controls (20.3 ± 8.8 *vs*. 8.2 ± 5.1 and 7.8 ± 3.0, P < 0.001), indicating proliferation-promoting factors in CSF after SAH. The PI was positively correlated with SAH volume (p = 0.025). For patients with lower SAH volume, patients with favorable outcome had a higher PI than those with poor outcome (20.8 ± 6.9 *vs*. 14.6 ± 4.3, p = 0.047). Using multivariable logistic regression analysis, the PI was a positive determinant for favorable outcome (odds ratio, 1.17; 95% confidence interval, 1.00 to 1.36) that more proliferation-promoting factors in CSF was associated with better functional outcome in SAH patients.

## Introduction

Stroke is one of the major causes of death and disability all over the world^1^. Many recent studies have focused on regenerative therapies for stroke^2,3^. Endogenous neural stem cells (NSCs), which are located mainly in the subventricular zone (SVZ) of the lateral ventricles and the subgranular zone (SGZ) of the hippocampal dentate gyrus, may also provide another attractive source for cell therapy^4^. Recently, many studies have shown that various kinds of brain injury, including stroke, may enhance neurogenesis at the SVZ and SGZ regions^5,6^. Stroke-induced neurogenesis may play an important role in functional recovery after stroke^7^. This concept has been demonstrated by conditional ablation of NSCs in adult mice diminishing poststroke motor and cognitive functional improvement and reduced synaptic connectivity^8,9^. However, the beneficial effects of neurogenesis have not been demonstrated in human studies, primarily because there is no available method to evaluate the neurogensis capacity in the brain of stroke patients.

Subarachnoid hemorrhage (SAH) is a subtype of stroke, which carries significant morbidity and mortality, with one-third of survivors suffering long-term physical and neurocognitive impairments^10^. In rodent brain, increased proliferation capacity of NSCs has been reported after SAH^11,12^. In human brain, SAH also induced the expression of NSC proliferation markers, indicating enhancement of neurogenesis^13^. To investigate the relationship between neurogenesis and SAH provides a chance not only to delineate the pathogenesis of the disease, but also to develop a potential treatment strategy, especially since there is still no effective therapy for SAH patients^14^.

In pathological conditions, injured brain may release some factors that diffuse to the SVZ and SGZ regions and stimulate neurogenesis^15^. Since the SVZ and SGZ areas are close to cerebral ventricle without the blood–brain barrier, the above factors may also appear in cerebrospinal fluid (CSF). Investigating CSF is thus a potential way to understand the relationships between neurogenesis and features of SAH. We have demonstrated that the NSC proliferation capacity is increased at the SVZ regions in a rat model of SAH at post-SAH 5–7 days^11^. Notably, the CSF collected from SAH rats during post-SAH 5–7 days can enhance the proliferating potentials of cultured rodent NSCs^11^. These findings imply the existence of proliferation-promoting factors in CSF of SAH rats. In this study, we tried to obtain CSF from SAH patients, measure the proliferation-promoting capacity of the CSF samples, and correlate this capacity to clinical features of SAH patients. We showed that the proliferation capacity of cultured NSCs promoted by CSF collected from SAH patients correlates well with disease severity and functional outcome.

## Results

### Optimization of condition using CSF to promote the proliferation capacity of NSCs

**Figure 1:**
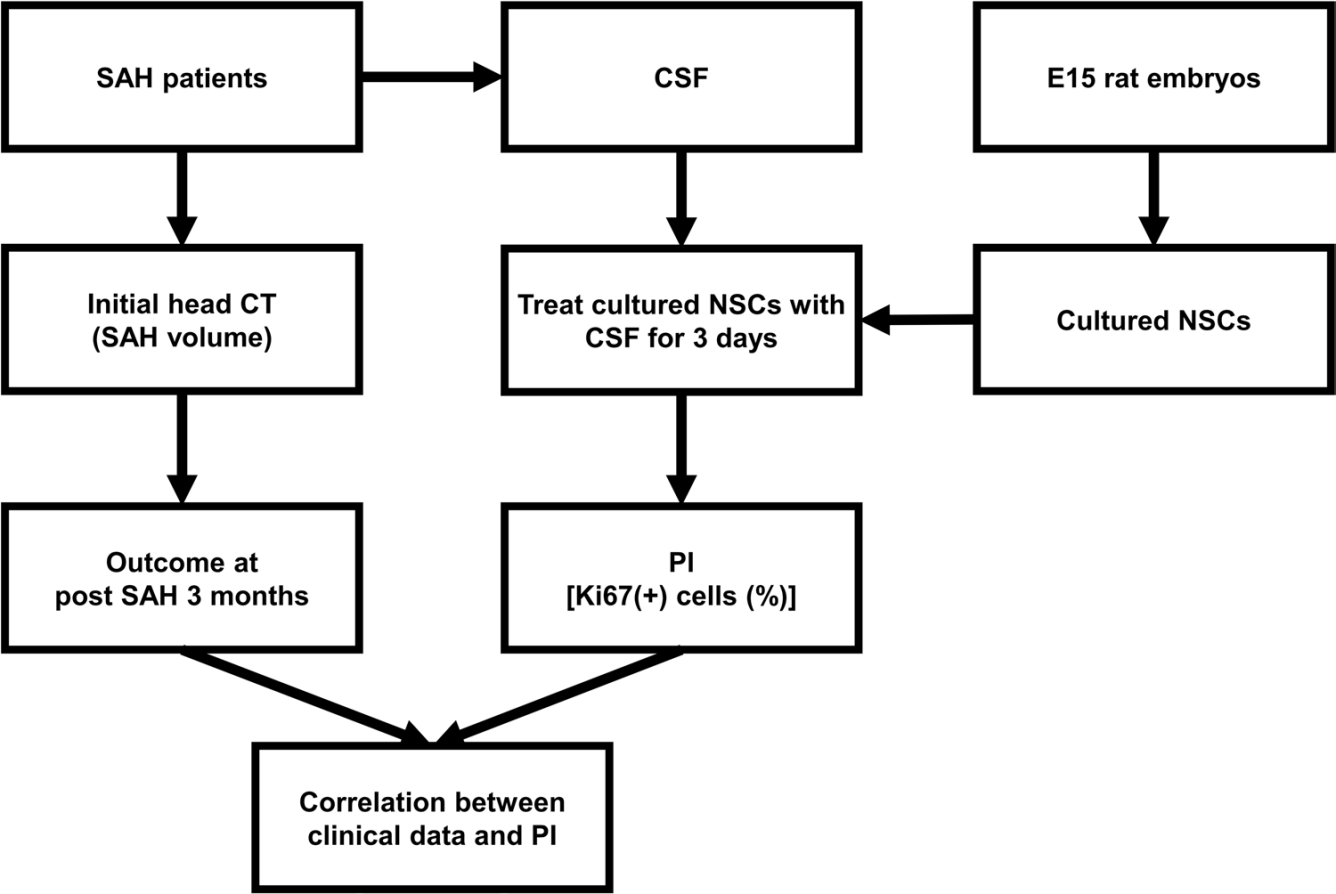
Study algorithm. Abbreviations: SAH, subarachnoid hemorrhage; CSF, cerebrospinal fluid; CT, computed tomography; NSC, neural stem cell; PI, proliferation index; E15, rat embryonic day 15.

We prospectively collected CSF samples from SAH patients (Fig. 1). The CSF was added to the culture medium of NSCs, which was obtained from rat embyronic day 15 (E15) fetal brain. The proliferation capacity of cultured NSCs with or without treatment was analyzed as the proliferation index (PI), which was defined by the percentage of NSCs having immunoreactivity to Ki-67, a proliferation marker^16^.

We previously demonstrated that CSF samples collected from SAH rats during post-SAH 5–7 days can enhance the proliferating capacity of cultured rodent NSCs in the neurosphere^11^. Therefore, we first tested the effects of human CSF samples collected from three SAH patients on post-SAH day 5 at different concentrations. The PI was higher in the SAH group than both normal pressure hydrocephalus (NPH) and untreated control groups in a dose-dependent manner (p < 0.05) (Supplementary Fig. S1). Since the proliferation promoting effects of CSF reached a plateau at the concentration of 0.5%, we applied this concentration in the experiments that follow.

**Figure 2:**
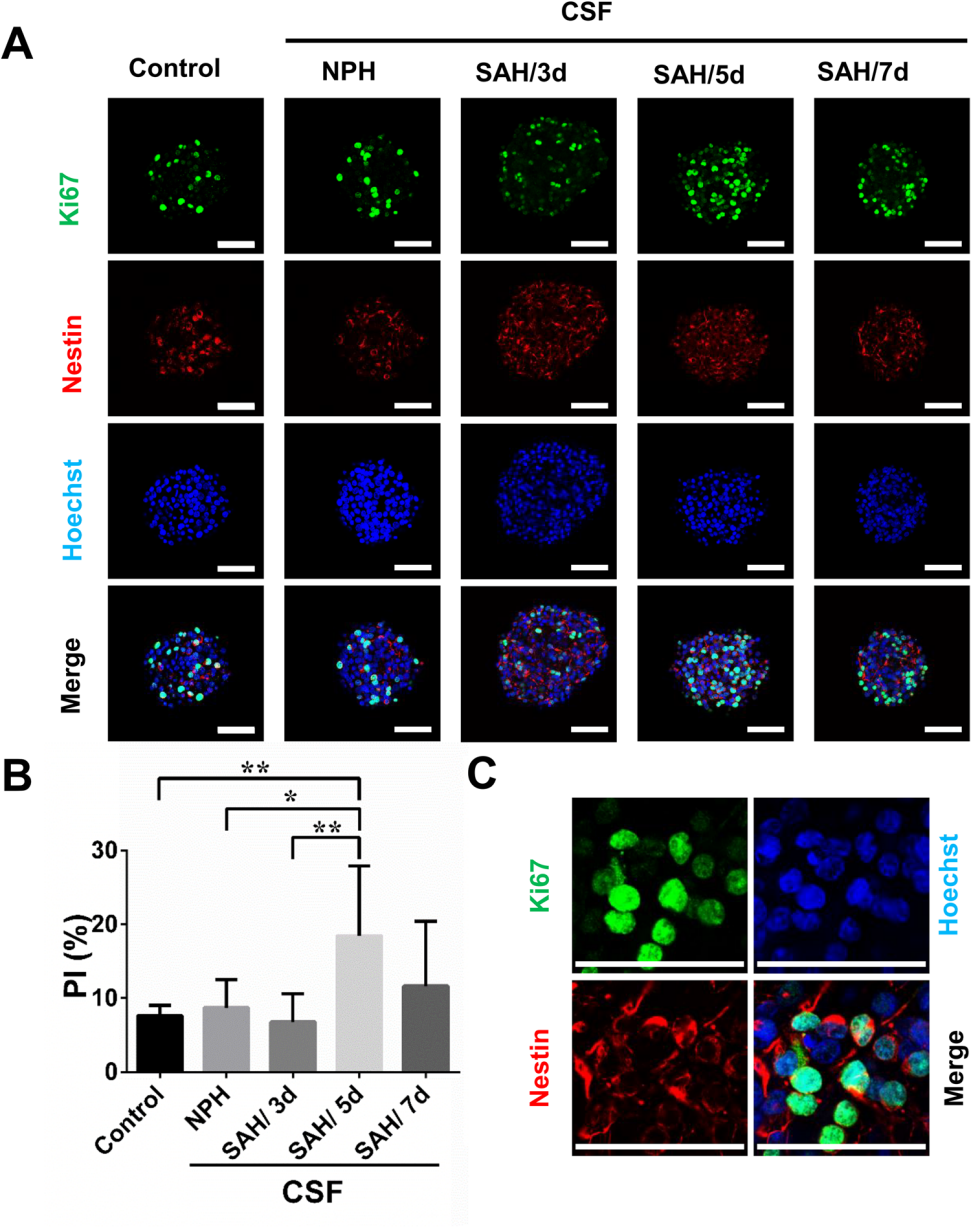
The effects of CSF samples collected at different post-SAH times on NSC proliferation. (**A**) The cultured NSCs without treatment (control) or treated with CSF samples collected from patients with NPH or SAH on day 3, 5, or 7 after onset were double immunostained with anti-Ki-67 (green) and anti-nestin (red) antibodies with Hoechst 33258 (blue) staining. Scale bar = 50 μm. (**B**) Comparison of the PI between different groups is shown. N = 6 for each group. (**C**) A high magnification image shows most Hoechst positive cells with or without Ki-67 immunoreactivity are also immunoreactive to nestin. Scale bar = 50 μm. *p < 0.05; **p < 0.01.

We further examined the effects of CSF samples, which were collected at different post-SAH times (days 3, 5, and 7) in six SAH patients. Only NSCs treated with CSF samples collected at 5 days post-SAH showed a higher PI as compared to those treated with CSF samples obtained from NPH patients and those without treatment (18.5 ± 9.5 *vs*. 8.7 ± 3.8 and 7.6 ± 1.4, P < 0.05) (Fig. 2A and B). The PI was also higher in NSCs treated with CSF samples collected on post-SAH day 5 than on day 3 (18.5 ± 9.5 *vs*. 6.8 ± 3.8, P < 0.01). We thus collected the CSF samples on day 5 post-SAH in all other patients. We also used BrdU method and cell viability assay kit (Cell Counting Kit-8, CCK8) to analyze the NSC proliferation, and found similar results that NSCs treated with CSF samples (n = 6) collected at 5 days post-SAH showed higher proliferation capacity as compared to those treated with CSF samples obtained from NPH patients and those without treatment (BrdU: 12.1 ± 2.6 *vs*. 3.3 ± 1 and 4.8 ± 1.1, P < 0.05; CCK8: 0.85 ± 0.07 *vs*. 0.64 ± 0.09 and 0.53 ± 0.18, P < 0.05) (Supplementary Figs S2 and S3). Using immunocytochemical analysis, most of the cells (90–95%) in neurospheres were immunoreactive to nestin (Fig. 2C), indicating the cells we analyzed were mainly NSCs.

### High proliferation index in treatment with CSF collected from SAH patients

The demographic data and clinical features of 36 SAH patients (age, 62 ± 13 years) are summarized in Supplementary Table S1. The SAH was caused by aneurysm rupture in all patients (two with dissecting aneurysm). Twenty patients (56%) had aneurysm located at anterior circulation and 16 (44%) at posterior circulation. The mean initial Glasgow Coma Scale (GCS) score was 11 ± 4 and the mean SAH volume was 19 ± 14 mL. Intraventricular hemorrhage (IVH) developed in 23 of 36 patients (63.9%) and vasospasm occurred in 21 patients (58.3%). All of the patients received aneurysmal clipping (n = 33) or endovascular embolization (n = 3) within 1 day after SAH onset except for 4 patients (2, 3, 4, and 6 days after onset without rebleeding before clipping or embolization). The median modified Rankin Scale (mRS) score accessed at post-SAH 3 months was 3 (0–6).

**Figure 3:**
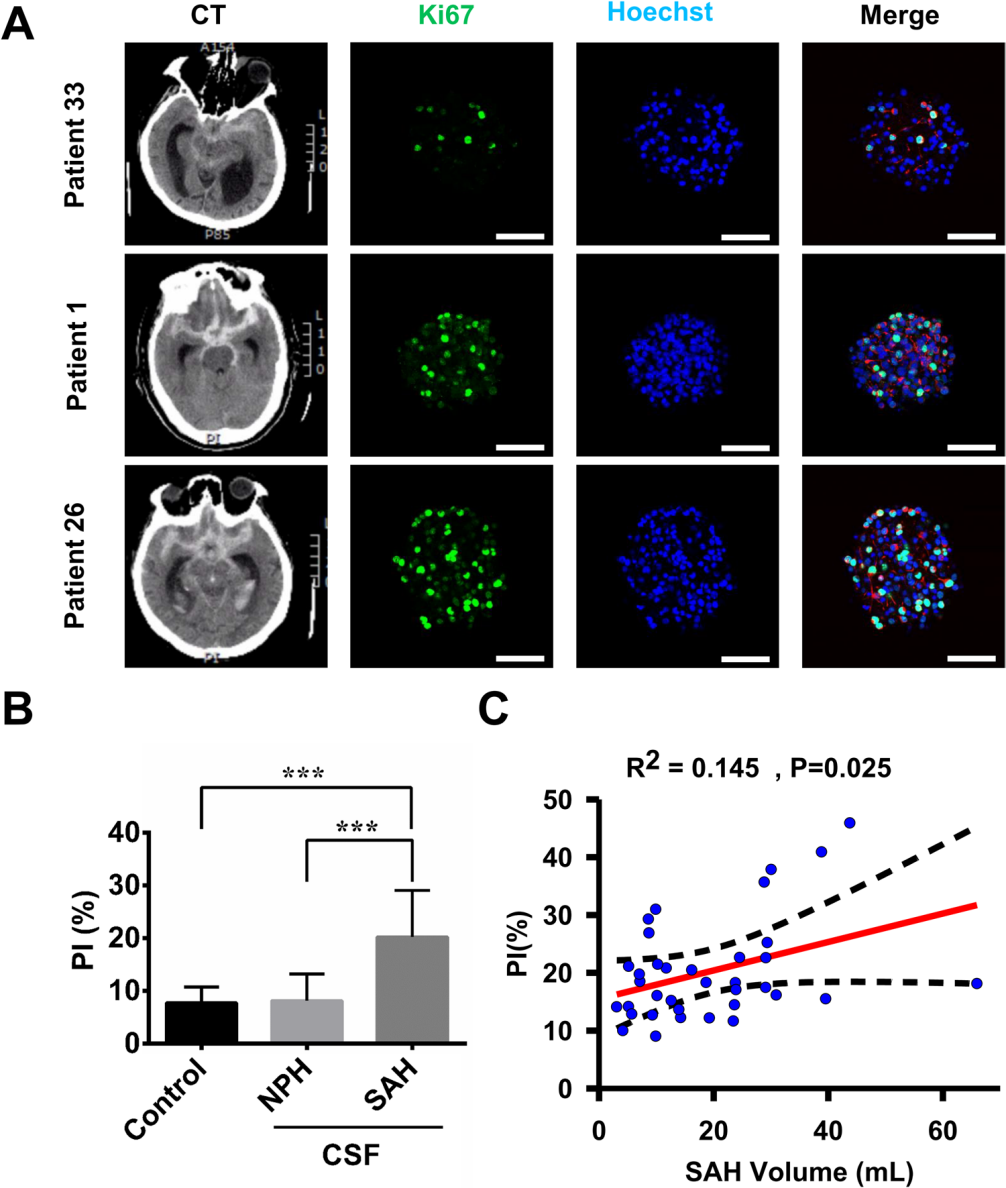
The effects of CSF samples collected at post-SAH day 5 on NSC proliferation. (**A**) The cultured NSCs without treatment (control) or treated with CSF samples collected from patients with NPH or SAH were double immunostained with anti-Ki-67 (green) and anti-nestin (red) antibodies with Hoechst 33258 (blue) staining. Initial head CT scan shows SAH in three patients (Patients 33, 1, and 26) with different severity (SAH volume of 9.9, 24.5, and 43.8 mL, respectively). The PI values in these three patients were 9.0, 22.6, and 45.9, respectively. Scale bar = 50 μm. (**B**) Comparison of the PI between different groups is shown, including control (n = 6), NPH (n = 6), and SAH (n = 36) groups. ***p < 0.001. (**C**) Correlation between the SAH volume and PI in 36 patients with SAH. p = 0.025 using linear regression analysis. The red solid line is the regression line and dashed lines are 95% confidence limits

The NSCs treated with CSF samples collected from 36 SAH patients on day 5 post-SAH showed a higher PI as compared to those treated with CSF samples obtained from six NPH patients and those without treatment (20.3 ± 8.8 *vs*. 8.2 ± 5.1 and 7.8 ± 3.0, P < 0.001) (Fig. 3A and B). The PI was then correlated with clinical information such as patient age, initial SAH volume, initial GCS, the existence of IVH, the presence of post-SAH vasospasm, and the location of aneurysm (Fig. 1). The PI values were positively correlated with SAH volume (Pearson’s r = 0.145; p = 0.025) (Fig. 3A and C). On the other hand, the PI values were not significantly correlated with age, gender, initial GCS, presence of IVH or vasospasm, or location of aneurysm (Supplementary Fig. S4).

### High PI associated with good post-SAH outcome

**Figure 4:**
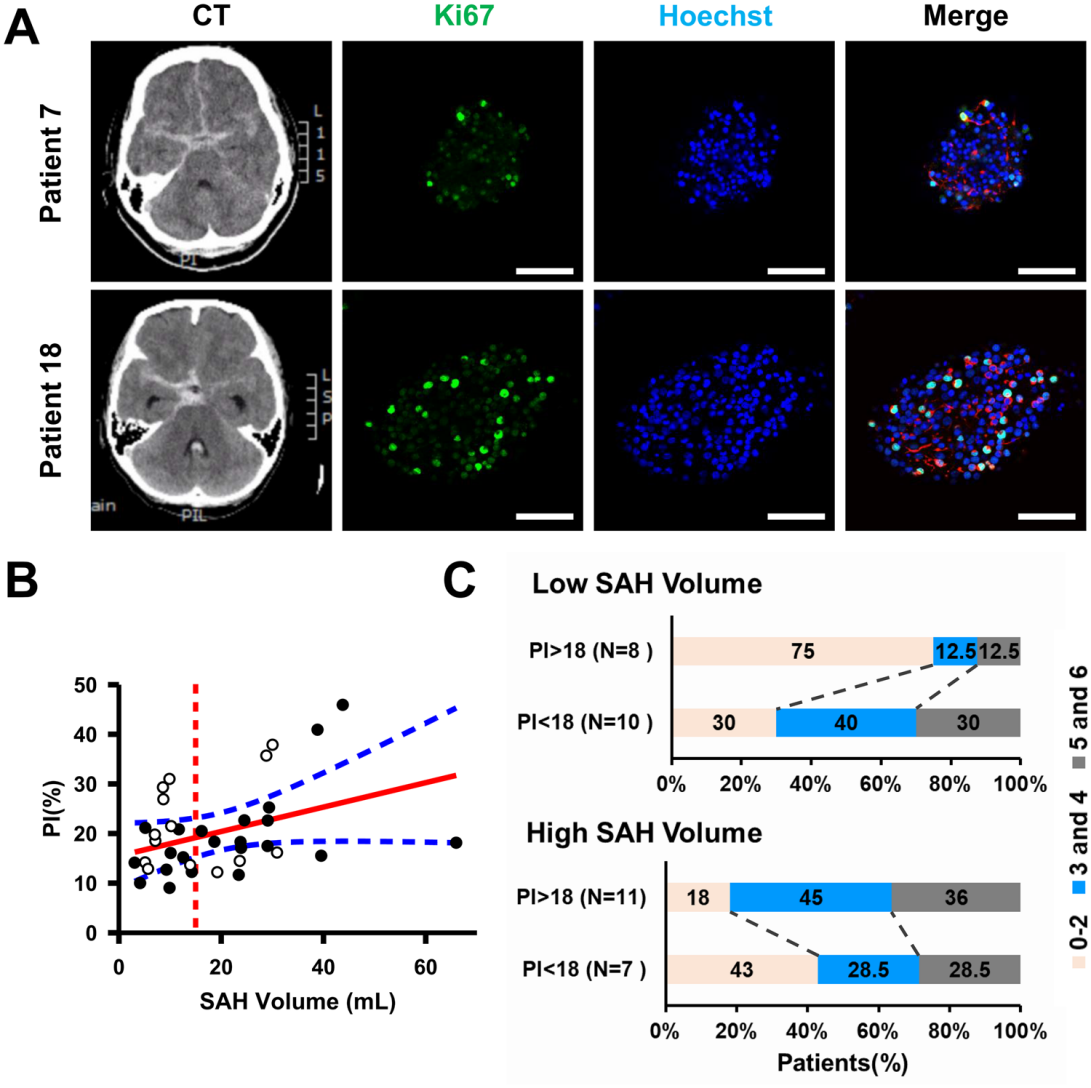
The relationship between the PI and post-SAH outcome. (**A**) The cultured NSCs treated with CSF samples collected from SAH patients were double immunostained with anti-Ki-67 (green) and anti-nestin (red) antibodies with Hoechst 33258 (blue) staining. Initial head CT scan shows SAH in two patients (Patients 7 and 18) with similar severity (SAH volume of 9.7 and 9.9 mL, respectively). The PI values in these two patients were 12.7 and 31.0, respectively; the mRS scores at post-SAH 3 months were 3 and 1, respectively. Scale bar = 50 μm. (**B**) The outcome of SAH patients according to the SAH volume and PI. White spots indicate the patients with favorable outcome (mRS < 3) and black spots indicate poor outcome. The red solid line is the regression line, blue dashed lines are 95% confidence limits, and the red dashed line marks the SAH volume at 15 mL. N = 36. (**C**) The percentage of patients with mRS of 0-2, 3-4, and 5-6 at post-SAH 3 months according to PI values in low and high SAH volume groups. The low or high SAH volume is defined as the SAH volume at the subarachnoid cistern less or more than 15 mL, respectively.

The overall correlation between the PI and mRS at post-SAH 3 months was not significant (Pearson’s r = 0.002; p = 0.75) (Supplementary Fig. S4). However, in patients with lower SAH volume (less than 15 mL in the subarachnoid cistern), a higher PI seemed to be associated with favorable outcome (mRS < 3) (Fig. 4A and B). Since the PI was correlated with SAH volume, which was potentially associated with outcome, we stratified SAH patients into two groups according the SAH volume in the subarachnoid cistern: low SAH volume (<15 mL) and high SAH volume (>15 mL). The median PI value of 36 patients was 18.6; therefore, we set the cutoff value for high and low PI at 18. In the low SAH volume group (n = 18), six of eight patients (75%) with high PI had favorable outcome while only three of ten patients (30%) with low PI had favorable outcome (Fig. 4C). On the other hand, in the high SAH volume group (n = 18), there was no obvious association between PI and outcome.

We further compared the features of patients with favorable and poor outcome (Table 1). In the low SAH volume group, patients with favorable outcome had a higher initial GCS score (13.3 ± 3.0 *vs*. 8.9 ± 3.9; p = 0.007) and a higher PI (20.8 ± 6.9 *vs*. 14.6 ± 4.3; p = 0.047) than those with poor outcome. Age, gender, SAH volume, aneurysm location, and presence of IVH or vasospasm were not significantly different between the groups. In the high SAH volume group, patients with favorable outcome had a higher proportion of males (80% *vs*. 15.4%; p = 0.022) than those with poor outcome, while other parameters including the PI were not significantly different between the groups.

**Table 1:** Comparison between patients with favorable and poor outcome by SAH volume.

	Low SAH volume			High SAH volume		
	Favorable Outcome (n = 9)	Poor Outcome (n = 9)	P-value	Favorable Outcome (n = 5)	Poor Outcome (n = 13)	P-value
Age (year)	57.8 ± 13.2	60.3 ± 12.3	0.66	56.0 ± 16.5	67.9 ± 9.6	0.14
Gender (men)	1 (11.1)	0 (0)	1	4 (80)	2 (15.4)	0.022
SAH volume (mL)	8.5 ± 2.7	8.7 ± 3.5	0.63	26.5 ± 5.0	31.3 ± 13.2	0.73
Initial GCS	13.3 ± 3.0	8.9 ± 3.9	0.007	12.0 ± 5.0	9.3 ± 3.1	0.098
IVH	4 (44)	7 (78)	0.34	3 (60)	9 (69.2)	1
Vasospasm	4 (44)	5 (56)	1	3 (60)	9 (69.2)	1
Location (posterior)	4 (44)	6 (67)	0.64	2 (40)	4 (30.8)	1
Proliferation Index	20.8 ± 6.9	14.6 ± 4.3	0.047	23.3 ± 12.4	22.6 ± 9.9	0.52

Values are mean ± standard deviation except gender, IVH, vasospasm, and location; number (percentage).

SAH indicates subarachnoid hemorrhage; GCS, Glasgow coma scale; IVH, intraventricular hemorrhage.

The low or high SAH volume is defined as the SAH volume at subarachnoid cistern less or more than 15 ml, respectively.

The posterior location of aneurysm includes vertebral or basilar arteries, posterior cerebral artery, posterior communicating artery, and posterior inferior cerebellar artery.

Favorable outcome, mRS = 0, 1, or 2.

Using univariate logistic regression analysis, only initial GCS (odds ratio [OR], 1.46; 95% confidence intervals [CI], 1.12 to 1.90) was a positive determinant for favorable outcome (Table 2). Using multivariable logistic regression analysis, the GCS (OR, 1.56; CI, 1.13 to 2.17) and PI (OR, 1.17; CI, 1.00 to 1.36) were positive determinants for favorable outcome. The SAH volume showed a trend to be a negative determinant (OR, 0.91; CI, 0.81 to 1.01) for favorable outcome. Adding other variables such as age, presence of vasospasm or IVH in the model showed similar results (Supplementary Table S2).

**Table 2:** Univariate and Multivariable analyses of determinants for favorable outcome in patients with subarachnoid hemorrhage.

	OR	95% CI	P-value
Univariate			
Age in year	0.95	0.89 to 1.01	0.088
SAH volume	0.95	0.89 to 1.01	0.13
Initial GCS	1.46	1.12 to 1.90	0.006
Presence of IVH	0.38	0.09 to 1.53	0.17
Presence of vasospasm	0.57	0.15 to 2.23	0.42
Aneurysm at posterior circulation	0.90	0.23 to 3.47	0.88
Proliferation index	1.03	0.96 to 1.11	0.43
Multivariable			
SAH volume	0.91	0.81 to 1.01	0.083
Initial GCS	1.56	1.13 to 2.17	0.007
Proliferation index	1.17	1.00 to 1.36	0.046

CI indicates confidence interval; SAH, subarachnoid hemorrhage; GCS, Glasgow coma scale; IVH, intraventricular hemorrhage; OR, odds ratio.

The posterior circulation includes vertebral or basilar arteries, posterior cerebral artery, posterior communicating artery, and posterior inferior cerebellar artery.

The model of multivariable analysis includes SAH volume, initial GCS, and the proliferation index.

## Discussion

Endogenous neurogenesis might contribute to the repair process following brain damage after stroke based on studies in mice which show ablation of neurogenesis impedes motor function recovery after cerebral ischemia^9,17^. However, the relationship between neurogenesis and post-stroke outcome has rarely been demonstrated in humans. After stroke, injured brain may release some factors to stimulate endogenous neurogenesis and these factors may also appear in CSF^15^. Therefore, investigating CSF is a potential way to understand the relationships between neurogenesis and features of stroke. We chose a stroke subtype, SAH, for this pilot study because lumbar drain is a routine management procedure for our SAH patients and therefore we could easily obtain the CSF samples in clinical practice. Here, we showed that the CSF from SAH patients enhanced the proliferation capacity of cultured rat NSCs in a severity-dependent manner, which indicates the existence of proliferation-promoting factors in CSF after SAH. The proliferation capacity positively correlated with clinical outcome, which may imply that more proliferation-promoting factors in CSF is associated with better functional outcome in SAH patients. Therefore, this study for the first time demonstrated the importance of NSC proliferation on the functional outcome of stroke patients, focusing on SAH.

Treatment with post-SAH CSF on cultured NSCs showed that only the CSF samples collected on day 5 post-SAH, and not on day 3 or day 7, can enhance the proliferation capacity of NSCs. Our findings were in accordance with those of previous studies which showed that the proliferation capacity is enhanced at the SVZ regions in a rat model of SAH on days 5–7 post-SAH and CSF collected from SAH rats during days 5–7 post-SAH can enhance NSC proliferation^11,12^. Since fresh blood containing red blood cells (RBCs) and plasma would appear in CSF of patients with SAH, we also treated the cultured NSCs with human RBCs and plasma to rule out their contributions to NSC proliferation. The PI was not increased in treatment with lytic or non-lytic RBCs (Supplementary Fig. S5) or plasma (Supplementary Fig. S6). Therefore, it is likely that post-SAH brain produces certain factors around 5 days after the onset of SAH which can promote the proliferation capacity of NSCs. Blood-derived neurotrophic factor (BDNF), fibroblast growth factor 2 (FGF-2), insulin-like growth factor 1 (IGF-1), vascular endothelial growth factor (VEGF), and epidermal growth factor (EGF) are important factors contributing to post-insult activation of neurogenesis^15,17^. In our preview study, BDNF concentration in CSF paralleled the temporal change in NSC neurogenesis after SAH in a rodent model^11^. BDNF is a potent modulator capable of regulating the functions of neurogenesis^18^, and might play a key role in post-SAH proliferation of NSCs. In addition, exosomes released from stem cells and some micro RNA, such as miR17–92 cluster and miR-124a are also able to enhance neurogenesis post ischemic stroke^19–21^. The exact proliferation promoting factors in CSF samples collected on day 5 post-SAH are still unclear and require further investigation.

Regarding the mechanisms of SAH-induced production and release of proliferation promoting factors, the hemorrhage-related neuro-inflammatory reactions and high intracranial pressure might play a role since SAH volume is positively associated with the proliferation promoting capacity of post-SAH CSF. Notably, previous studies have shown that neuro-inflammation is able to enhance endogenous neurogenesis^22^. SAH may be complicated by vasospasm leading to brain ischemia^23^, which may also induce neurogenesis in post-insult brain^5,6,24^. However, we did not detect a correlation between the PI and the presence of vasospasm, making the possibility of brain ischemia less likely to be a major contributory mechanism for NSC proliferation.

Using multivariable logistic regression with control of the variables including age, infarct volume, initial GCS, and presence of IVH or vasospasm, the proliferation promoting capacity of post-SAH CSF positively independently correlated with clinical outcome, which indicates that more proliferation promoting factors in CSF is associated with better functional outcome in SAH patients. It is reasonable that with the higher post-SAH production of proliferation promoting factors in CSF, the endogenous NSCs at the SVZ and SGZ of post-SAH brain would receive stronger stimulation to undergo cell proliferation, as we observed in the culture system. Post-stroke neurogenesis can promote tissue remodeling and neural repair, which have been demonstrated in the rodent model of cerebral infarct^25^. Therefore, in this study, our findings supported the beneficial role of endogenous NSC proliferation on functional recovery in stroke patients, focusing on SAH. Enhancement of endogenous neurogenesis is thus a reasonable and potential therapeutic strategy for SAH treatment. In addition to high initial GCS associated with favorable outcome, a high PI value might be another independent predictor of favorable outcome for SAH patients. We also found that favorable outcome was associated with high proliferation promoting capacity of post-SAH CSF only in the low SAH volume group, not in the high volume group. This means that while more proliferation promoting factors in CSF may enhance post-SAH functional improvement, the benefits are not strong enough to overcome the pernicious effects of massive SAH. In the future, when neurogenesis-related therapy is available for SAH patients, analyzing the NSC proliferation-promoting capacity of CSF might help us to select appropriate cases (according to PI and SAH volume) for further treatment that patients with low PI values and relatively lower SAH volume might be a good candidate to receive certain management to enhance the endogenous NSC proliferation.

This study had some limitations. First, we could not obtain CSF from healthy volunteers and therefore used CSF from NPH patients as a control instead. However, we cannot guarantee that the results obtained with CSF samples from NPH patients are totally normal. Second, although we clearly demonstrated the existence of proliferation promoting factors in CSF after SAH, we did not identify the specific factors in this study. In addition, we only enrolled patients with SAH but not cerebral infarct or intracerebral hemorrhage, which are more common subtypes of stroke^1^. Future studies should try to identify the proliferation-promoting factors in CSF and extend the investigation to other types of stroke. Third, *in vitro* NSC proliferation and actual *in vivo* neurogenesis may be different. However, in our previous rodent study, we found that the neurogenesis at the SVZ region is enhanced in a rat model of SAH on days 5–7 post-SAH; the CSF collected from SAH rats during days 5–7 post-SAH can promote NSC proliferation^11^. The result may suggest that *in vitro* NSC proliferation is likely associated with *in vivo* post-SAH neurogenesis.

In summary, we provided a novel method using human CSF to study the relationship between NSC proliferation capacity and functional outcome in patients with SAH. The CSF from SAH patients enhanced the proliferation capacity of cultured NSCs, indicating the presence of neurogenesis promoting factors in CSF after SAH. The proliferation capacity positively correlated with clinical outcome, which implies that more proliferation-promoting factors in CSF is associated with better functional outcome in SAH patients. This study thus supports the rationale to enhance the proliferation capacity of NSCs as a reasonable therapeutic strategy for stroke treatment, especially for SAH.

## Methods

### Patients

We prospectively recruited 36 adult patients with SAH from National Taiwan University Hospital who received lumbar drainage for CSF diversion. Initial head computed tomography (CT) was performed all within 1 day except for one patient (within 2 days) after the onset of SAH. We only included SAH patients with modified Fisher grading score of 3 and 4, which are defined as thick SAH (>1 mm in depth) without and with IVH, respectively^26^. The patients were all monitored in the neurointensive care unit and treated with the standard protocol which consisted of resuscitation, early surgical or endovascular obliteration of the aneurysm, standard management of intracranial pressure, neurointensive care, and aggressive medical or endovascular therapy for vasospasm if present^14^. All patients received nimodipine for prevention of vasospasm.

The CSF was collected via lumbar drain on day 5 after SAH. We obtained CSF on days 3, 5, and 7 after SAH in the first six patients for initial analysis of the optimal time to collect the CSF. The CSF samples were immediately centrifuged at 900 g at 4 °C for 20 min before being divided into suitable aliquots and snap-frozen at −80 °C within 30 min. We also collected CSF from six patients with NPH to use as a control. The research was approved by the National Taiwan University Hospital Committee of Human Research and conducted in accordance with human ethics regulations (No. 201605042RINB). Written informed consent was obtained from the patients or from the next of kin of patients who had decreased consciousness levels.

### Isolation and culture of neural stem cells

NSCs were obtained from pregnant Wistar rats at the gestational age of 15 days according to a protocol previously described^11,27^. First, embryos were removed from the rat, and the embryonic cerebral cortices were dissected out, washed, triturated, and cultured in the complete media containing Dulbecco’s Modified Eagle Media (DMEM)/F-12 (Gibco, Pascagoula, MS) supplemented with 1% N2 supplement (Gibco), 20 ng/ml basic fibroblast growth factor (bFGF), and 1% antibiotic solution absence of serum. Cultures were incubated at 37 °C in a humidified atmosphere and 5% CO2 for 6 days by which time primary neurospheres would form. Neurospheres were then cultured on 24-welled tissue culture polystyrene (TCPs, Costar, San Diego, CA) at 200 ± 20 neurospheres/cm^2^ in the DMEM/F12 medium supplemented with 1% N2 supplemented, 1% antibiotic-antimycotic solution (Gibco), and different concentration (0.25%, 0.5% and 1%) of CSF for 3 days without changing the culture medium. In addition to immunocytochemistry study, the Cell Counting Kit-8 (CCK-8) (Sigma-Aldrich, St. Louis, Missouri) was used to assess the NSC proliferation capacity. Equal amount of NSC was cultured with or without 0.5% CSF. After 3 days, CCK-8 solution was added to culture medium and NSCs were incubated at 37 °C for additional 4 hours. Optical density (OD) was then determined at a wave-length of 450 nm.

The animal experimental procedures were approved by the National Taiwan University Institutional Laboratory Animal Care committee and the Utilization Committee (No. 20160109). All procedures met the requirements of the Animal Welfare Protection Act of the Department of Agriculture, Executive Yuan, Taiwan.

### Immunocytochemistry

In immunocytochemical characterization, cells were fixed with ice-cold methanol for 20 min, blocked with 1% bovine serum albumin for 30 minutes at room temperature, and then incubated with primary antibodies overnight at 4 °C. For BrdU labeling, NSCs were pretreated with BrdU (Roche, Basel, Switzerland) at 10 μM for 3 days, fixed with methanol, and then treated with 4% Formaldehyde/1% Triton X100 for 10 min and 1 M HCl for 10 min before blocked. The primary antibodies used in this study were rabbit anti-Ki67 polyclonal antibody (a proliferation marker; 1:300, Abcam, Cambridge, MA), mouse anti-BrdU monoclonal antibody (a proliferation marker; 1:500, Cell Signaling technology, Danvers, MA), and mouse anti-nestin monoclonal antibody (a NSC marker; 1: 300, Merck Millipore, Billerica, MA). After wash, cells were incubated with secondary antibodies and Hoechst 33258 (diluted in PBS) for 2 hours at room temperature. The secondary antibodies used in the experiment were Alexa Fluor 488-conjugated goat anti-ribbit IgG and CyTM3-conjugated donkey anti-mouse IgG (1:200; Jackson ImmunoResearch, West Grove, PA). The immunostained cells were visualized by confocal LSM880 microscope (Carl Zeiss AG, Oberkochen, Germany) with the optical thickness of 2 μm. For each condition, we analyzed at least three neurospheres with at least three planes for each neurosphere. The sub-PI of each plane was calculated as the areas with Ki-67 (or BudU) immunoreactive cells divided by the areas with Hoechst 33258-stained cells; the areas were determined automatically using MetaMorph software (Leica Microsystems GmbH, Wetzlar, Germany). All the sub-PI values were then averaged to get a final PI for each condition.

### SAH severity and outcome analysis

The severity of SAH was determined by the volume of blood that appeared in the subarachnoid cisterns (basal, ambient, sylvian, suprasellar, and frontal interhemispheric cisterns) on initial axial head CT (thickness, 0.5 cm). The area of hemorrhage on each film was measured manually using software of ImageJ. The median SAH volume in the subarachnoid cistern of 36 patients was 15.2 ml; therefore, we set the cutoff value for high and low SAH volume at 15 ml. The location of aneurysm is stratified into anterior circulation (internal carotid artery, anterior or middle cerebral arteries, anterior communicating artery, and anterior choroidal artery) and posterior circulation (vertebral or basilar arteries, posterior cerebral artery, posterior communicating artery, and posterior inferior cerebellar artery). A follow-up head CT was performed around 1 week post-SAH in all patients to detect potential new intracerebral hypodense lesions, which indicate post-SAH cerebral infarction. An additional head CT with CT angiography was performed if neurological deterioration developed or when vasospasm was suspected during post-SAH care within 2 weeks. The existence of vasospasm was defined as the presence of post-SAH cerebral infarct detected with head CT or vasospasm detected with CT angiography. The functional outcome was evaluated using the mRS at 3 months after onset of SAH. An mRS score less than 3 (able to look after own affairs without assistance) was defined as a favorable functional outcome^28^. The researcher who measured the PI was blinded to the clinical condition of SAH patients and the researchers who analyzed the SAH volume or accessed the mRS were blinded to the PI values.

### Statistical analysis

The results are reported as mean ± standard deviation (SD). To compare the PI between groups, the Kruskal-Wallis test with the Mann-Whitney test as post-hoc analysis was used. We correlated the PI value with clinical data, including age, gender, initial GCS, SAH volume, IVH, vasospasm, location of aneurysm, and mRS using linear regression analysis. To analyze the outcome, the Mann-Whitney test and Fisher’s exact test were used to determine the differences between groups for continuous data and categorical data, respectively. A logistic regression model was used to analyze the determinants of favorable outcome. The model included SAH volume, initial GCS, PI value, age, presence of IVH, and presence of vasospasm. A p-value of less than 0.05 was considered statistically significant. The SPSS statistical software (version 10.0, SPSS) was used for the statistical analyses.

## Electronic supplementary material

Supplementary Information
