# Supplementary material for: The Proliferation Capacity of Cultured Neural Stem Cells Promoted by CSF Collected from SAH Patients Correlates to Clinical Outcome
Source: Sci Rep. 2018 Jan 18;8:1109. doi: 10.1038/s41598-018-19371-5 (PMC5773507; doi:10.1038/s41598-018-19371-5)
Supplement: Supplementary file 1 — Supplementary Information [file 41598_2018_19371_MOESM1_ESM.pdf]

The Proliferation Capacity of Cultured Neural Stem Cells Promoted by CSF Collected from SAH Patients Correlates to Clinical Outcome

Yun-An Chen[1], Kuo-Chuan Wang[2], Der-Zen Liu[3], Tai-Horng Young[1,*], Li-Kai Tsai[4,*]

[1]Institute of Biomedical Engineering, College of Medicine and College of Engineering, National Taiwan University, Taipei, Taiwan

[2]Department of Surgery, National Taiwan University Hospital and National Taiwan University College of Medicine, Taipei, Taiwan;

[3]Graduate Institute of Biomedical Materials and Tissue Engineering, College of Biomedical Engineering, Taipei Medical University, Taipei, Taiwan

[4]Department of Neurology and Stroke Center, National Taiwan University Hospital and College of Medicine, Taipei, Taiwan

Correspondence should be addressed to:

*Li-Kai Tsai, M.D., Ph.D.; Department of Neurology, National Taiwan University Hospital, No. 7, Chung-Shan South Road, Taipei, 100, Taiwan

Tel: 886-2-23123456#23476; Fax: 886-2-2341-8395; E-mail:milikai@ntuh.gov.tw

*Tai-Horng Young, Ph.D.; Institute of Biomedical Engineering, College of Medicine and College of Engineering, National Taiwan University, Taipei, 100, Taiwan

Tel.: +886 2 23123456 ext 81455; Fax: +886 2 23940049; thyoung@ntu.edu.tw

Supplementary Tables

Supplementary Table S1. Demographic and clinical data of patients with subarachnoid hemorrhage

No.	Age (Year)	Sex	Aneurysm Location	Initial GCS	IVH	SAH Volume (mL)	Management	vasospasm	mRS
1	71	F	Acom	8	Y	24.5	clipping	N	5
2	57	F	Acom	14	Y	16.2	clipping	Y	3
3	52	M	Acom	14	N	23.8	clipping	Y	3
4	55	F	Pcom	6	Y	29.1	clipping	Y	5
5	72	F	distal ICA	12	N	7.1	clipping	N	1
6	75	F	Acom	7	Y	39.6	clipping	Y	4
7	54	F	Acom	10	N	9.3	clipping	N	3
8	67	F	Acom	12	N	29.4	clipping	Y	3
9	58	M	Acom	15	N	30.9	clipping	Y	2
10	65	F	VA	10	Y	12.6	Embolization	N	3
11	72	F	Pcom	6	Y	29.1	clipping	Y	3
12	53	F	Pcom	7	Y	3.1	clipping	Y	3
13	85	M	BA	6	Y	66.0	Embolization	Y	6
14	58	F	MCA	15	N	5.2	clipping	Y	0
15	70	F	ACA	13	Y	18.7	clipping	Y	5
16	32	F	Pcom	15	N	7.1	clipping	Y	2
17	68	F	Pcom	9	Y	4.2	clipping	Y	5
18	58	F	Ach A	15	Y	9.9	clipping	N	1
19	59	F	MCA	14	N	23.6	clipping	Y	2
20	60	F	Pcom	10	Y	23.5	clipping	N	3
21	67	F	MCA	10	N	23.9	clipping	N	6
22	42	F	BA	7	Y	11.7	clipping	Y	3
23	64	F	PICA	6	Y	8.7	clipping	N	2
24	64	M	distal ICA	14	Y	30.1	clipping	N	1
25	77	F	Acom	9	N	38.8	clipping	Y	3
26	75	F	Acom	6	Y	43.8	clipping	N	6
27	66	F	MCA	7	N	14.3	clipping	N	3
28	73	F	Pcom	10	Y	10.1	clipping	N	5
29	74	F	Pcom	13	Y	10.2	clipping	N	2
30	71	M	Pcom	3	Y	28.8	clipping	Y	2
31	28	M	Pcom	14	Y	19.3	clipping	N	0
32	45	F	VA	8	Y	5.2	Embolization	Y	6
33	77	F	ACA	10	Y	9.9	clipping	Y	5

34	47	F	Pcom	14	N	8.6	clipping	Y	1
35	50	F	Acom	15	N	13.9	clipping	Y	2
36	65	M	Ach A	15	Y	5.7	clipping	N	1

Acom indicates anterior communicating artery; Pcom, posterior communicating; ICA, internal carotid artery; ACA, anterior cerebral artery; PCA, posterior cerebral artery; MCA, middle cerebral artery; Ach A, anterior choroidal artery; VA, vertebral artery; BA, basilar artery; mRS, modified Rankin scale; IVH, intraventricular hemorrhage; GCS, Glasgow coma scale; SAH, subarachnoid hemorrhage; Y, yes; N, no; M, male; F, female

Supplementary Table S2. Multivariable analysis of determinants for favorable outcome using different models

	Model 1		Model 2		Model 3	
	OR	95% CI	OR	95% CI	OR	95% CI
Age in year	0.97	0.90 to 1.05				
SAH volume	0.91	0.81 to 1.02	0.91	0.82 to 1.02	0.90	0.79 to 1.01
Initial GCS	1.55	1.10 to 2.17	1.59	1.13 to 2.23	1.52	1.09 to 2.13
Proliferation index	1.18	1.00 to 1.40	1.16	1.00 to 1.36	1.17	1.01 to 1.37
Presence of vasospasm			0.58	0.09 to 3.57		
Presence of IVH					0.53	0.07 to 3.88

CI indicates confidence interval; SAH, subarachnoid hemorrhage; GCS, Glasgow coma scale; IVH, intraventricular hemorrhage; OR, odds ratio.

The model 1 includes age, SAH volume, initial GCS, and the proliferation index.

The model 2 includes SAH volume, initial GCS, the proliferation index, and the presence of vasospasm.

The model 3 includes SAH volume, initial GCS, the proliferation index, and the presence of IVH.

Supplementary Figures

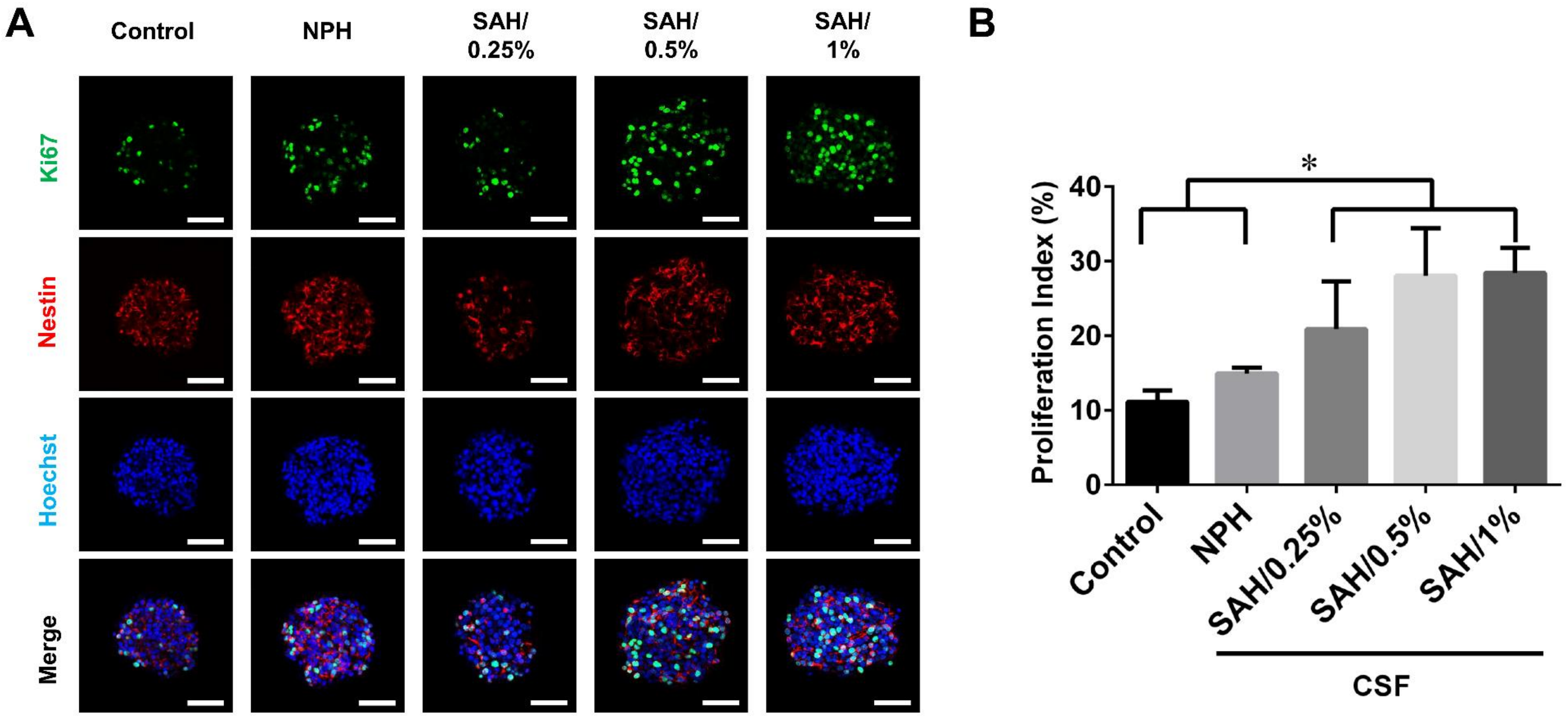

Supplementary Fig. S1. The effects of CSF samples at different concentrations on NSC proliferation. (A) The cultured NSCs without treatment (control) or treated with CSF sampled collected from patients with NPH or SAH on day 5 after onset with concentrations of 0.25%, 0.5% and 1% were double immunostained with anti-Ki67 (green) and anti-nestin (red) antibodies with Hoechst 33258 (blue) staining. Scale bar = 50 µm. (B) Comparison of the PI between different groups was shown. N=3 for each group. Means ± SD. *, $p < 0.05$, Kruskal-Wallis test and Mann-Whitney test.

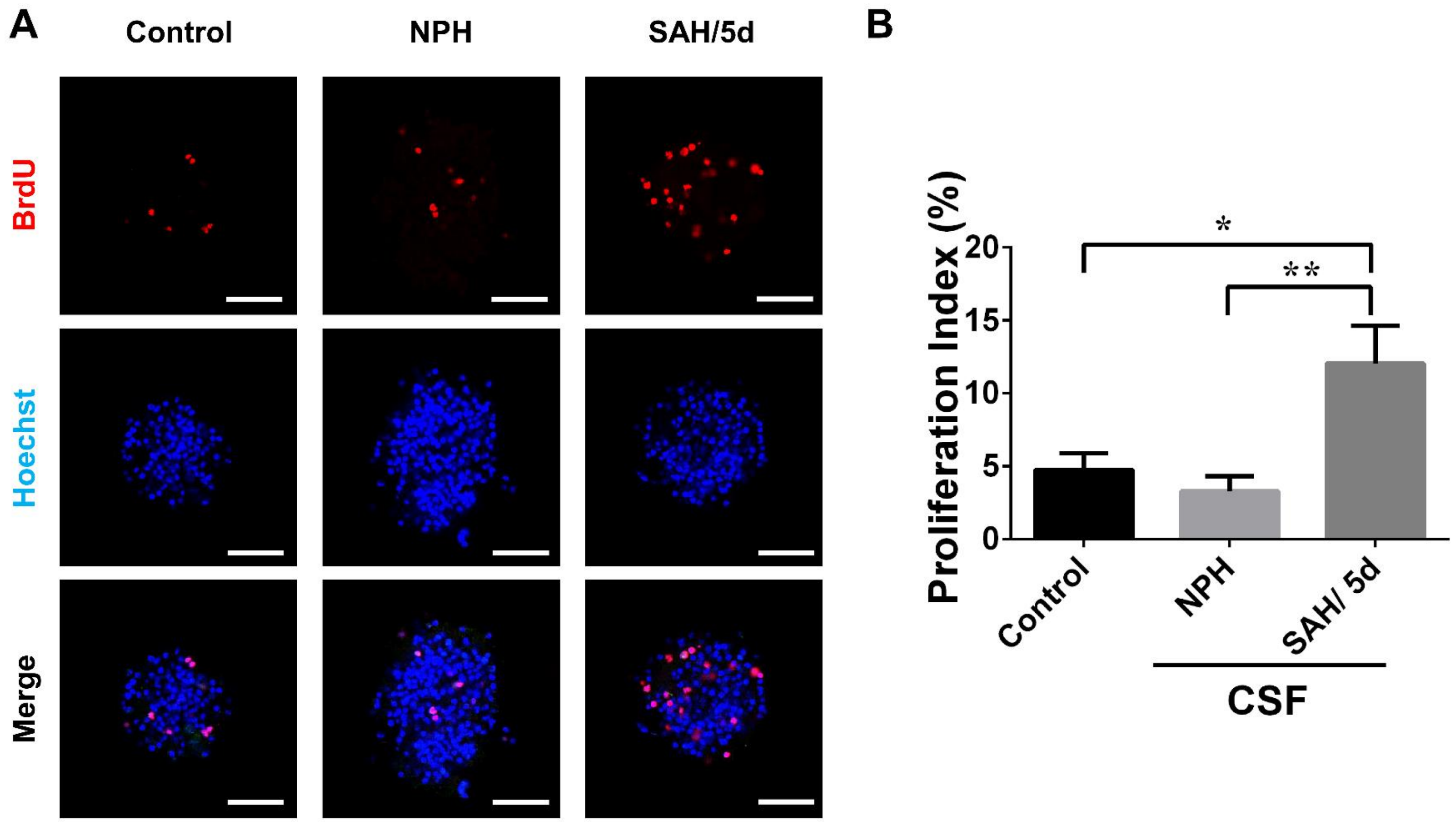

Supplementary Fig. S2. The effects of CSF samples on NSC proliferation. (A) The cultured NSCs without treatment (control) or treated with CSF sampled collected from patients with NPH or SAH on day 5 after onset with concentrations of 0.5% were immunostained with anti-BrdU (red) antibodies with Hoechst 33258 (blue) staining. Scale bar = 50 μm. (B) Comparison of the PI between different groups was shown. N=6 for each group. Means ± SD. *, $p < 0.05$; **, $p < 0.01$, Kruskal-Wallis test and Mann-Whitney test.

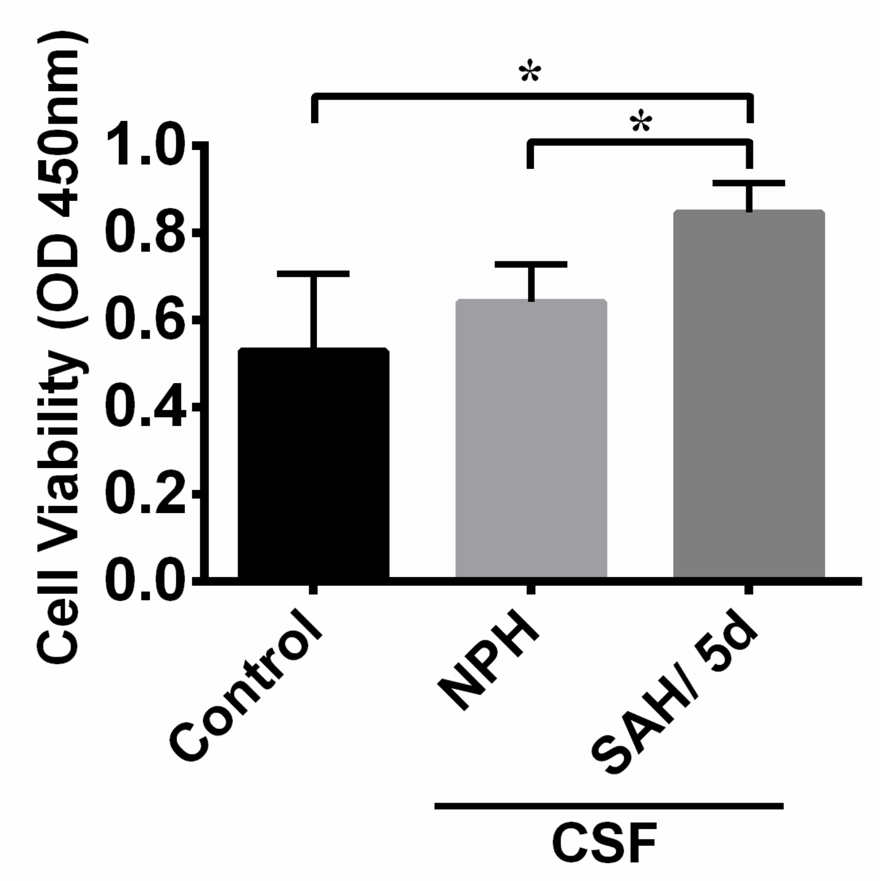

Supplementary Fig. S3. The effects of CSF samples on NSC proliferation. Comparison of the cell viability between different groups was shown. N=6 for each group. Means ± SD. *, $p < 0.05$, Kruskal-Wallis test and Mann-Whitney test.

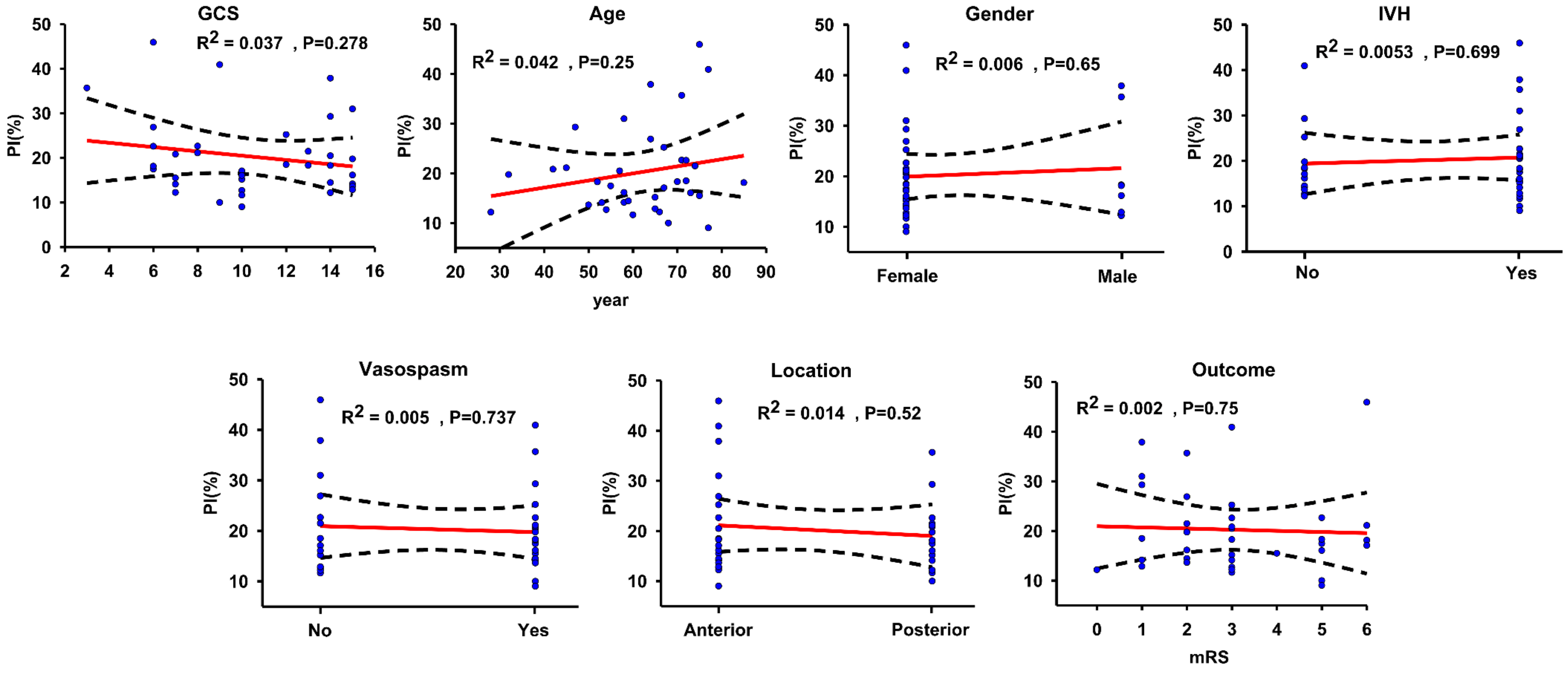

Supplementary Fig. S4. Correlation of the PI to variable clinical features, including initial Glasgow Coma Scale (GCS), age, gender, presence of intraventricular hemorrhage (IVH) or vasospasm, location of the ruptured aneurysm, and the modified Rankin scale (mRS) at post-SAH 3 months. The location of aneurysm is stratified into anterior circulation (internal carotid artery, anterior or middle cerebral arteries, anterior communicating artery, and anterior choroidal artery) and posterior circulation (vertebral or basilar arteries, posterior cerebral artery, posterior communicating artery, and posterior inferior cerebellar artery). The red solid line is the regression line and dash lines are 95% confidence limits.

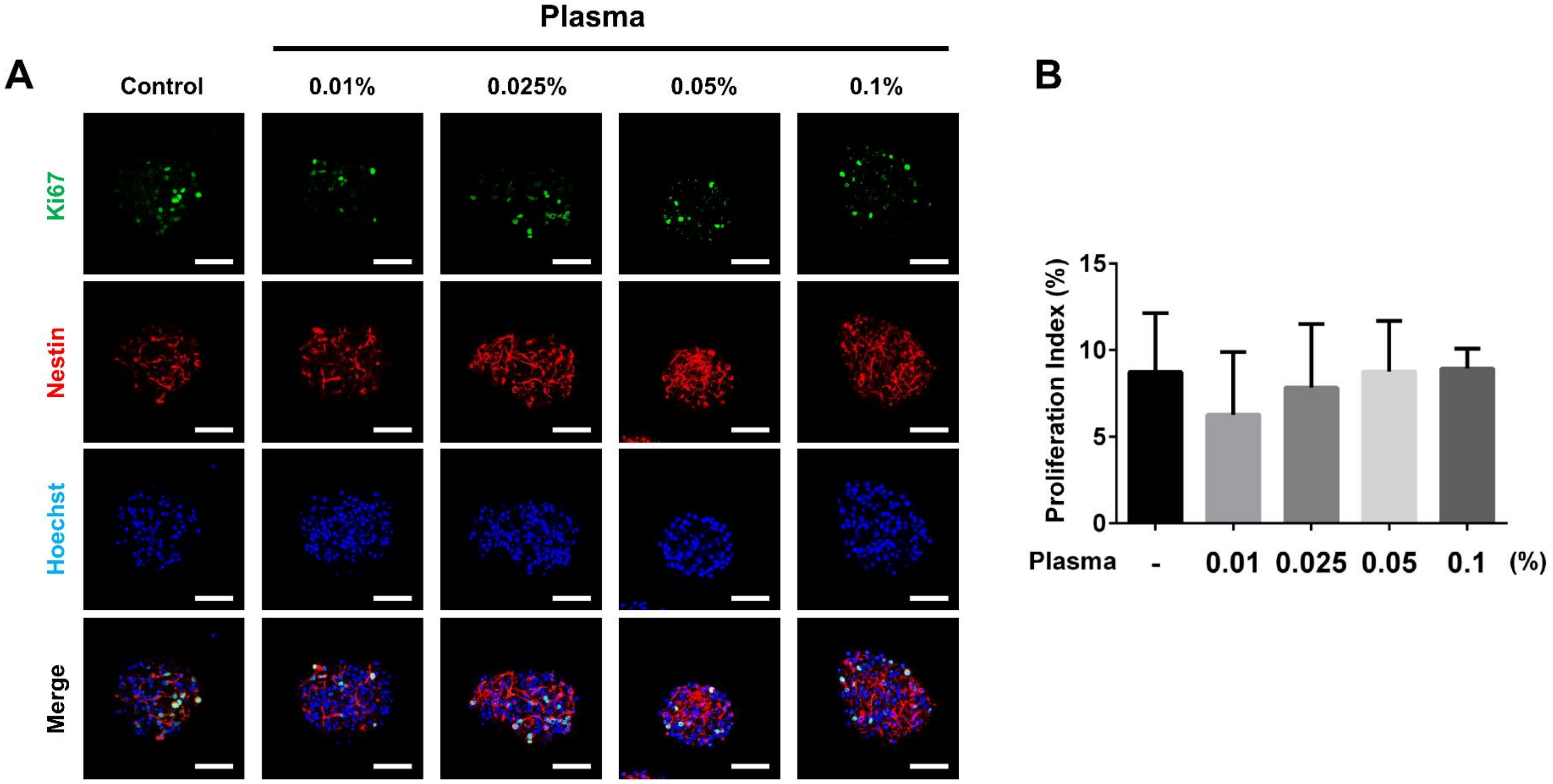

Supplementary Fig. S5. The effects of red blood cells (RBCs) on NSC proliferation. (A) The cultured NSCs treated with RBC with or without lysis using water pretreatment were double immunostained with anti-Ki67 (green) and anti-nestin (red) antibodies with Hoechst 33258 (blue) staining. The fresh blood was obtained from a healthy volunteer and the sample was centrifuged at 3000 rpm for 10 minutes. The plasma and RBCs were then collected separately. For RBC lysate, the RBC sample was added in equal volume of water, followed by trituration through a 1000-μl pipette to break the RBCs. Scale bar = 50 μm. (B) Comparison of the PI between different groups was shown. Means ± SD. Kruskal-Wallis test.

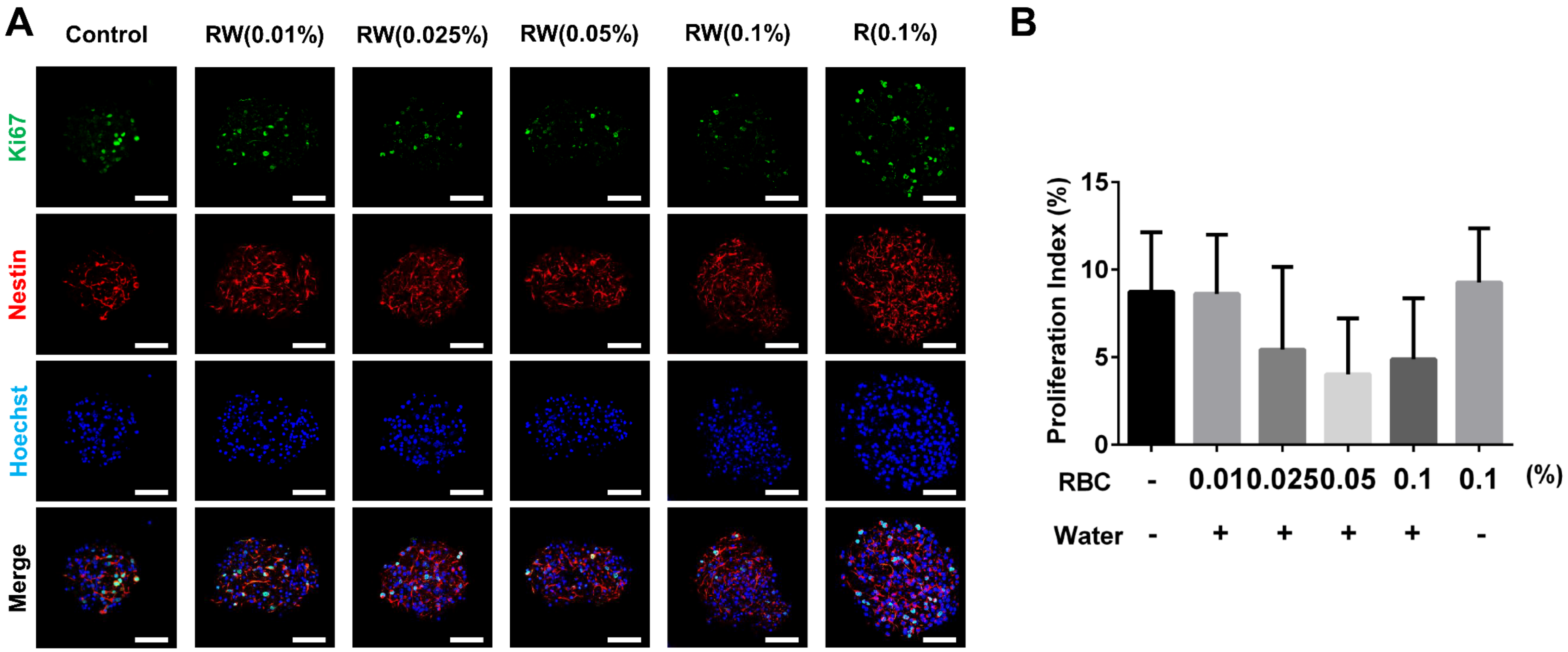

Supplementary Fig. S6. The effects of plasma on NSC proliferation. (A) The cultured NSCs treated with or without plasma at different concentrations were double immunostained with anti-Ki67 (green) and anti-nestin (red) antibodies with Hoechst 33258 (blue) staining. Scale bar = 50 µm. (B) Comparison of the PI between different groups was shown. Means ± SD. Kruskal-Wallis test.
